# COVID-19 Pandemic Data Modeling in Pakistan Using Time-Series SIR

**DOI:** 10.1155/2022/6001876

**Published:** 2022-06-28

**Authors:** Muhammad Taimoor, Sajid Ali, Ismail Shah, Fred Roland Muwanika

**Affiliations:** ^1^Department of Statistics, Quaid-i-Azam University, Islamabad 45320, Pakistan; ^2^School of Statistics, Makerere University, P.O. Box 7062, Kampala, Uganda

## Abstract

Pakistan is currently facing the fourth wave of the deadly coronavirus, which was first reported in Wuhan, China, in December 2019. This work utilizes the epidemiological models to analyze Pakistan's COVID-19 data. The basic susceptible, infected, and recovered (SIR) model is studied assuming Bayesian and time-series SIR (tSIR) approaches. Many studies have been conducted from different perspectives, but to the best of our knowledge, no study is available using the SIR models for Pakistan. The coronavirus incubation period has been set to 14 days across the globe; however, this study noticed that the assumption of 14 days is not suitable for Pakistan's data. Furthermore, on the basis of *R*_0_, we infer that COVID-19 is not a pandemic in Pakistan, as it was in other nations, such as the United States, India, Brazil, and Italy, among others. We attribute this to the best strategy adopted by the Government of Pakistan to minimize the burden of COVID-19 cases in Pakistani hospitals. It is also noticed that the posterior-based SIR (pSIR) model with uniform prior to*R*_0_ and Poisson distribution (of log-likelihood) provides better results as compared to other distributions. From time-series SIR (tSIR), we observed that the value of the reporting rate (*ρ*) is less than 1 which means that cases are underreported.

## 1. Introduction

There are numerous types of coronaviruses, some of which cause severe threats to human life. Coronavirus gets its name from its shape—“corona” means “crown.” The virus's top layers are coated with spike proteins, which act as a crown. The fatal COVID-19 disease started in 2019 which is a respiratory sickness caused by coronavirus. It first appeared in Wuhan, China, near the end of December 2019. The scientists initially treated it as pneumonia, but it spread at an alarming rate all over the world and became the first pandemic of the twenty-first century with a high reproductive rate. The International Committee on Taxonomy of Viruses named it severe acute respiratory syndrome coronavirus-2 [1]. However, on March 11, 2021, the World Health Organization (WHO) named it coronavirus disease-19 (COVID-19). According to WHO, there have been 532,201,219 confirmed cases, with 6,305,358 deaths worldwide, till June 6, 2022.

According to data from recent examinations, the severity of the disease differs epidemiologically by race, gender, and age [2]. It is almost two years, and no one knows how long it will stay around. Scientists are working hard to control it, but no particular solution has been found yet. The majority of patients infected with COVID-19 have mild-to-moderate symptoms and recover without any additional therapy. The researchers now believe that COVID-19 is transmitted mostly by droplets, and virus particles are dispersed into the atmosphere when an infectious agent coughs, laughs, talks, sings, sneezes, or breaths out. The most typical symptoms of COVID-19 include exhaustion, high temperature, sinus infection, discomfort, conjunctivitis, sore throat, diarrhea, headache, taste and odor loss, discoloration of the fingers or toes, lack of oxygen, chest pain, and speech and movement impairments. The WHO advised the following precautions against COVID-19:

- Hands should be cleaned often with soap or any alcohol-based liquid for at least 20 seconds
- Keep a safe distance (minimum 2 meters) away from somebody coughing or sneezing
- When physical separation is not possible, use a mask
- Avoid touching your eyes, nose, or mouth
- Use tissue or bent elbow while coughing or sneezing
- If you are sick, stay at home and take care of yourself
- Seek medical help if you have a fever and cough or feel difficulty in breathing
- Masks can prevent others if someone has infection and vice versa

The first incidence of COVID-19 in Pakistan was recorded and reported by the Ministry of Health, Government of Pakistan, on February 26, 2020, in Karachi, Sindh. On the same day, Pakistan's Federal Ministry of Health confirmed another case in Islamabad. Within fifteen days, there were twenty confirmed cases out of 471 suspected cases. Sindh province had the greatest number, followed by Gilgit-Baltistan. All of the confirmed cases had traveled from Iran, Syria, or London, and the situation in Pakistan is still alarming. On February 12, 2020, Pakistan's Ministry of National Health Services, Regulation, and Coordination unveiled a plan titled “National Action Plan for Preparedness and Response to Corona Virus Disease Pakistan,” with the goal of controlling virus spread and strengthening country and community emergency preparedness to ensure a timely, convenient, and prompt solution for future occurrences. The government introduced various measures over time to control the spread of coronavirus in the country. It includes containment measures, border control, quarantine houses, countrywide smart lockdowns, cordoning off areas, testing and contact tracing, establishing a field epidemiology laboratory program with the help of WHO, implementation of SOPs, initiation of an awareness campaign, production of ventilators, and other economic measures [3–5].

If we consider the current COVID-19 pandemic and try to develop a model that predicts how this mortal disease will behave in the upcoming months in Pakistan, it is plausible to think how many of those susceptible can be infected by a single infectious person. Suppose a single infection-carrying person can infect three others who are in contact with him. Then, the model will be like *I*_*t*_ = *I*_0_^*t*^, where *I*_*t*_ is the total number of infected people at time *t* and *I*_0_^*t*^ is the initial number of infected people. This equation gives us the sudden increase in the disease and predicts that the epidemic will never end. Thus, if we use this single ordinary differential equation (ODE) to model the epidemic, it would not capture the real situation and will show totally different and coarse graphics as compared to the real situation. That is why the SIR model is preferred. The SIR model is time-dependent and depends upon the initial values.

Weiss [6] used the SIR model in public health, whereas Finkenstädt and Grenfell [7] used time-series SIR (tSIR) modeling for children's disease measles. Grenfell et al. [8] studied the dynamics of measles using the tSIR model with the aim of providing a suitable empirical and theoretical test to address the importance of noise versus nonlinearity and temporal predictability of population wealth. Katris [9] used the tSIR, autoregressive integrated moving average (ARIMA), feedforward artificial neural network, and adaptive regression splines for forecasting the outbreak of COVID-19 in Greece. They compared different countries using *R*_0_ and the mean absolute percentage error (MAPE). Pasquali et al. [10] introduced a beta distribution for the observation error in a SIR model to model the underdetection and removed individuals in the compartmental model for COVID-19.

Chen et al. [11] conducted a study on COVID-19 by assuming two scenarios, i.e., the time-wise prediction, time-dependent parameters (*β* and *γ*) and the undetectable number of infected individuals, to get a more accurate prediction using the time-dependent SIR (tSIR) model. Deo et al. [12] studied the transmission rate of COVID-19 in India using the tSIR model. They estimated that the total infections crossed 9 million with 1 million critical cases. They also estimated *R*_0_ for smart and full lockdowns in different phases. Postnikov [13] conducted a study on COVID-19 using the simple SIR model for parameter estimation and future predictions. Metcalf et al. [14] conducted a study related to rubella in Mexico to capture seasonality, stochasticity, and region-wise variation. Lavielle et al. [15] extended the SIR model to model the COVID-19 data taken from Johns Hopkins University on the basis of the daily confirmed, active, death, recovered, and cumulative number of cases for each compartment. They applied the extended model to several countries like Switzerland, Italy, and the US.

Bjornstad et al. [16] estimated the transmission rates of measles for England and Wales using the tSIR model. Deo and Grover [17] noticed that the unreported pathogens are more threatening than reported and quarantined ones. Their proposed model was susceptible-infected (quarantined/free)-removed-deceased (SI(Q/F)RD). The estimated values of *R*_0_ of undetected pathogens for California and Florida were 1.464 and 1.612, respectively. On the other hand, *R*_0_ for reported pathogens were 0.497 and 0.359 for respective states at the time of the study. Fang et al. [18] conducted a study on the contagion moral force of COVID-19 to imitate the dispersion of COVID-19 using the susceptible-exposed-infected-recovered (SEIR) model. Waris et al. [3] assessed COVID-19 in Pakistan using different factors like hospital capacity, isolation, quarantine, and vaccination facilities.

Brugnano et al. [19] studied the multiregional extension of the SIR model for the COVID-19 outbreak in Italy to capture the effect of misdiagnosed infected and recovered pathogens. They used the susceptible, diagnosed-infected, undiagnosed-infected, diagnosed-recovered, and undiagnosed-recovered (S*I*_2_*R*_2_) model. Ferrari et al. [20] conducted a study about the seasonal incidence change of measles in sub-Saharan Africa. They estimated seasonal/yearly variation by applying the tSIR model to 17-year-long time-series (monthly) data of Niamey (1986-2002). Althaus [21] estimated the basic reproductive number of the Ebola virus in West Africa. Li et al. [22] estimated the parameters of the stochastic SIR model using the maximum likelihood and Bayesian approaches with reference to media coverage of COVID-19.

There are different statistical and mathematical (deterministic) models that exist for epidemiological modeling. The existing models can also be used to predict the basic reproductive number of any pandemic like COVID-19. The SIR model, which is also known as the compartmental model, is used to predict the basic reproductive ratio. The SIR is the simplest form of the compartmental models, and all other models are the derivatives of this basic model. The SIR model has three different compartments, susceptible, infected, and recovered. The basic assumption of the traditional SIR model is that the infected (*I*) and susceptible (*S*) populations are mixed uniformly and that the overall population (*N*) remains constant throughout time [23]. Many studies, including time-series models for forecasting and mathematical models, are available for modeling the COVID-19 epidemic and for its prediction. Yousaf et al. [24] used the ARIMA model for forecasting COVID-19 in Pakistan which was the first study to make short-term forecasting about COVID-19 cumulative confirmed cases in Pakistan. The number of confirmed cases was increasing at a higher rate as compared to the number of recoveries. Contrary to their forecasted cases, the reality was totally different. Thus, we prefer to use compartmental modeling for the prediction of the basic reproductive number and forecasting purpose which is somehow performing better than statistical or time-series models used in epidemiological studies. Thus, the primary objective of the study is to predict the reproductive number and forecast COVID-19 cases in Pakistan using the SIR model. Further objectives of the study are to estimate the parameters (contact rate (*β*), recovery rate (1/*γ*), force of infection (*λ*), and reproductive number (*R*_0_)) of the model using Pakistan's COVID-19 data. In addition, the study investigates whether cases are underreported or overreported and decides whether it is a pandemic or not in Pakistan.

The rest of the study is categorized as follows.  Section 2 presents the simple SIR, posterior-based SIR, and tSIR models. Analyses using the tSIR, SIR, and pSIR are discussed in Sections 3–5.  Section 6 presents some concluding remarks and recommendations.

## 2. The SIR Model

The SIR model [1], introduced by Kermack and McKendrick in 1927, is a simple epidemiological model. This primary and simplest deterministic model is used for modeling the trend of epidemics (infectious diseases) and for obtaining its future predictions [25]. The population used in this model comprises three compartments which are as follows: (i) susceptible (*S*): the population not yet infected but can be infected by contact with an infected individual; (ii) infected (*I*): the number of persons who have been infected by the disease and can now transmit it; and (iii) recovered (*R*): the number of individuals who got infected but now have been shifted from the infected to the recovered compartment or are dead. Hence, *S* + *I* + *R* = *N*. The special feature of SIR is that it is used to find the basic reproductive number of a pandemic, denoted by *R*_0_. *R*_0_ basically tells us how many susceptible people can be infected (on average) by a single pathogen. If it is less than 1, we conclude that the pandemic will end very soon, and if it is greater than 1, then it is a pandemic and will take time to end. If *R*_0_ is greater than 3, the whole population will be infected [26].

The mathematical form of the SIR model is

(1) $$\begin{array}{c} \frac{dS}{dt}=-\beta S\left(t\right)I\left(t\right),\\ \frac{dI}{dt}=\beta S\left(t\right)I\left(t\right)-\gamma I\left(t\right),\\ \frac{dR}{dt}=\gamma I\left(t\right), \end{array}$$

where *dS*/*dt* + *dI*/*dt* + *dR*/*dt* = 0. The basic reproductive number *R*_0_ is computed by *R*_0_ = (*β*/*γ*)*N*.

### 2.1. Bayesian Approach to the SIR Model

In the Bayesian approach, the SIR model is slightly different from the simple SIR model as the Bayesian approach treats*R*_0_as a random variable, and one can assume negative binomial, Poisson, normal, or log-normal distribution as the prior distribution. A slight modification of the SIR model is given below.

(2) $$\begin{array}{c} P_{\inf}\frac{dS}{dt}=-SI\frac{R_{0}}{N},\\ P_{\inf}\frac{dI}{dt}=\left(\frac{R_{0}}{N}S-1\right)I,\\ P_{\inf}\frac{dR}{dt}=I. \end{array}$$

It is worth mentioning that the interpretation of the model is the same as that of the simple SIR model. This model has two parameters, *R*_0_ and infectious period (*P*_inf_). In this study, we use uniform distribution as a prior distribution of *R*_0_:

(3) $$\begin{array}{c} f\left(R_{0}\right)=\left\{\begin{array}{cc} \frac{1}{b-a}, & \text{for }R_{0}\in \left[a,b\right],\\ 0, & \text{otherwise}. \end{array}\right. \end{array}$$

### 2.2. Time-Series SIR Model (tSIR)

A time-series susceptible-infected-recovered (tSIR) model proposed by [7] is employed to study the dynamical behavior of the COVID-19 data. To this end, the data comprise two discrete variables, i.e., “infected cases” and “susceptible.” The SIR model can be expressed by the following three ODEs:

(4) $$\begin{array}{c} \frac{dS}{dt}=\xi P\left(t\right)-\frac{\lambda S\left(t\right)}{N}-\mu \left(t\right)S\left(t\right),\\ \frac{dI}{dt}=\frac{\lambda S\left(t\right)}{N}-\gamma I\left(t\right)-\mu \left(t\right)I\left(t\right),\\ \frac{dR}{dt}=\gamma I\left(t\right)-\mu \left(t\right)R\left(t\right), \end{array}$$

where *ξ* ∈ (0, 1), *ξP*(*t*) is the fraction of respiratory patients with SARS-2 infection, and *ξ* might be approximated as a constant near 1 (e.g., 0.9), but it will depend on the time in general. *λ* is the force of infection which can be expressed as *Iβ*/*N*, *μ* is the total number of deaths at time *t*, and *γ* is the recovery parameter. In the cyclical occurrences of the epidemic, the transmission rate *β* would vary with time. The traditional models give us estimates of each parameter against seasonal data. As we know, the tSIR model is tractable in the situations where seasonality occurs, but it also has some flaws. First, it takes only one main variable, “reported cases.” Second, there might be many underreported and overreported cases. Therefore, in this study, we use the “people having respiratory problems” to fulfill the requirements of the tSIR model. It is noticed from the literature that on a daily basis, almost 13,000 persons suffer from respiratory issues (https://www.aku.edu/news/Pages/News_Details.aspx?nid=NEWS-000849). Thus, to capture under- or overreporting, the tSIR model is modified as

(5) $$\begin{array}{c} S_{t+1}=P_{t+1}-S_{t}-I_{t+1}, \end{array}$$

(6) $$\begin{array}{c} E\left[I_{t+1}\right]=\beta_{t+1}S_{t}I_{t}^{\alpha }, \end{array}$$

where *P*_*t*+1_ and *I*_*t*+1_ are the one-day-ahead forecasted number of people having respiratory problems and reported cases, respectively. Similarly, *β*_*t*+1_ is the one-day-ahead forecasted contact rate given by the tSIR model. Under the tSIR framework, the R function “runtSIR” first fits a simple regression model between the cumulative reported cases and the cumulative number of people having respiratory problems. As there should be a linear relationship between reported cases and people with respiratory problems, from the slope of the fitted regression line, we can conclude that the cases are either underreported or overreported. We name this slope as *ρ*_*t*_, and it tells us whether the cases are under- or overreported. If the value of *ρ* is near 1, one may assume that cases are almost entirely reported, and if it is less than 1, one can conclude that cases are underreported. However, if its value is more than one, one can assume that instances are being reported excessively. The “runtSIR” function also gives us *S*_*t*_ and *β*_*t*_, susceptible dynamics with respect to time *t* and contact rate of the single pathogen over time *t*, respectively. After taking the expectation of equations (5) and (6), one can get the following log-linear equation:

(7) $$\begin{array}{c} \log I_{t+1}=\log\beta_{t+1}+\log\left(Z_{t}+\bar{S}\right)+\alpha \log I_{t}, \end{array}$$

where *Z*_*t*_ is the residual of the fitted regression model and *α* is the estimated homogeneity parameter by using the generalized linear model (GLM). $\bar{S}$ is the mean number of susceptible people of the overall series, and *α* is a parameter which describes the intensity of the epidemic. Furthermore, the “runtSIR” fits the above log-linear relationship and resimulates the SIR model (forward and backward) by using the estimated parameters.

## 3. Analysis Using the tSIR Model

**Figure 1:**
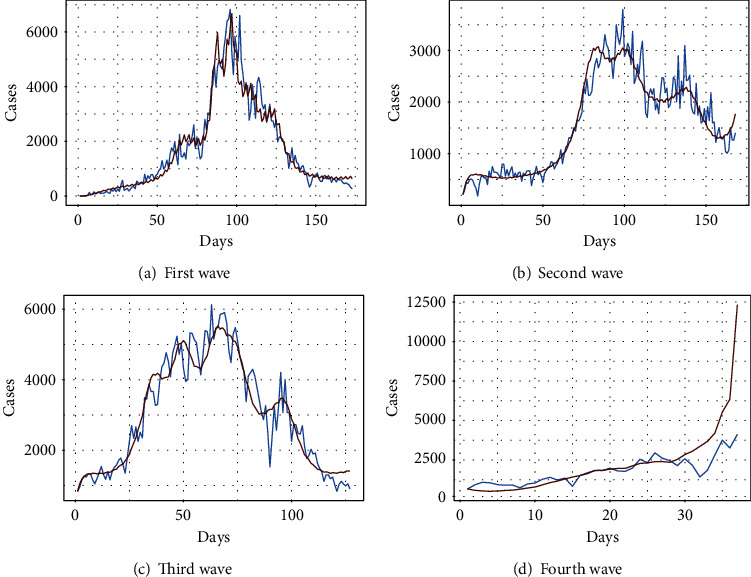
Wave-wise fitted tSIR model.

Figures 1(a)–1(d) present the behavior of the wave-wise fitted tSIR model for the first four waves of COVID-19 in Pakistan using the optimum values of parameters (listed in Table 1). In particular, the blue curve shows the actual number of daily reported cases while the gray curve presents the behavior of the cases using the tSIR model. On the basis of prior lag daily cases, it is noticed that the first wave is better fitted by the tSIR. However, in the second wave, there is a small amount of uncertainty between the fitted number and the observed number of instances. There is a variation in the third wave, but it appears to be a better match overall. However, when we look at the fourth wave, a significant increase in the fitted curve is noticed, which indicates that the disease may pose a serious threat in the upcoming days.

**Figure 2:**
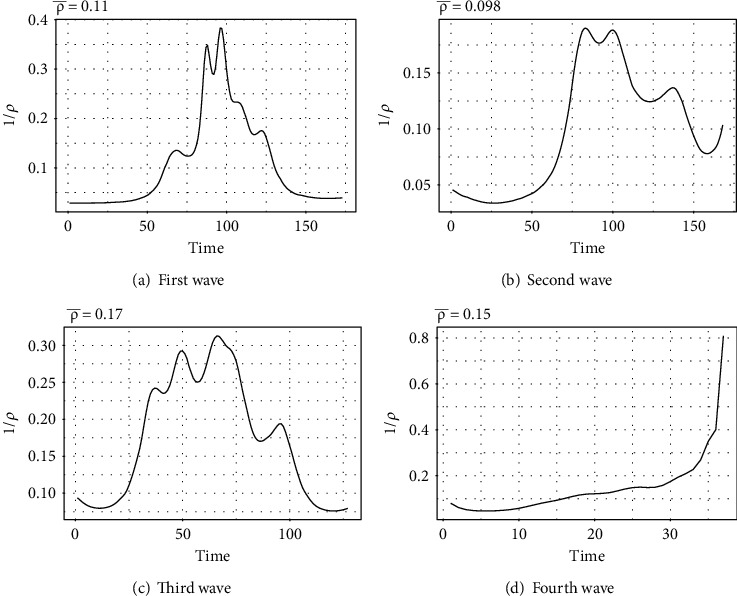
Wave-wise behavior of parameter *ρ* estimated by the tSIR model.

Figure 2 shows the wave-wise behavior of $\bar{\rho }$ of COVID-19 data for Pakistan.

**Figure 3:**
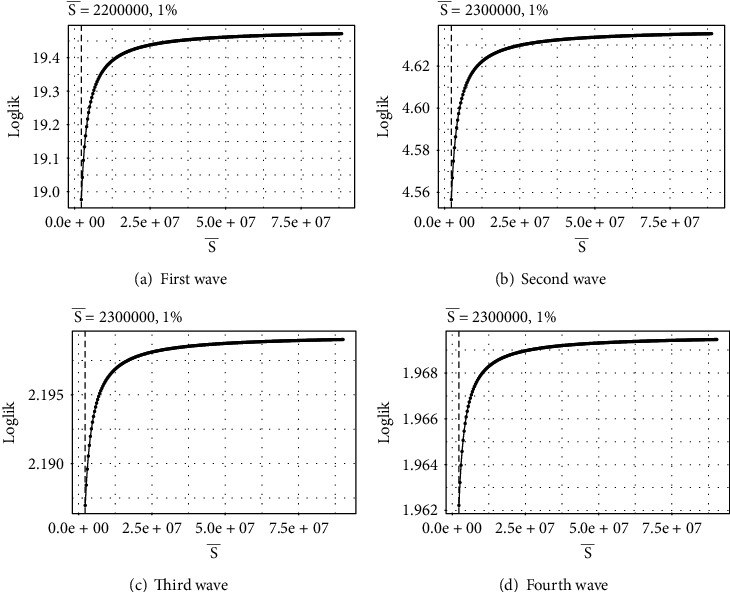
Wave-wise upcoming graphical behavior of the total number of susceptible people.

Figure 3 reports the daily total number of susceptible people estimated by the tSIR model for four waves of COVID-19, where $\bar{S}$ is the mean number of susceptible people. To depict this figure, initially, 1% of the population is taken as the susceptible population.

**Figure 4:**
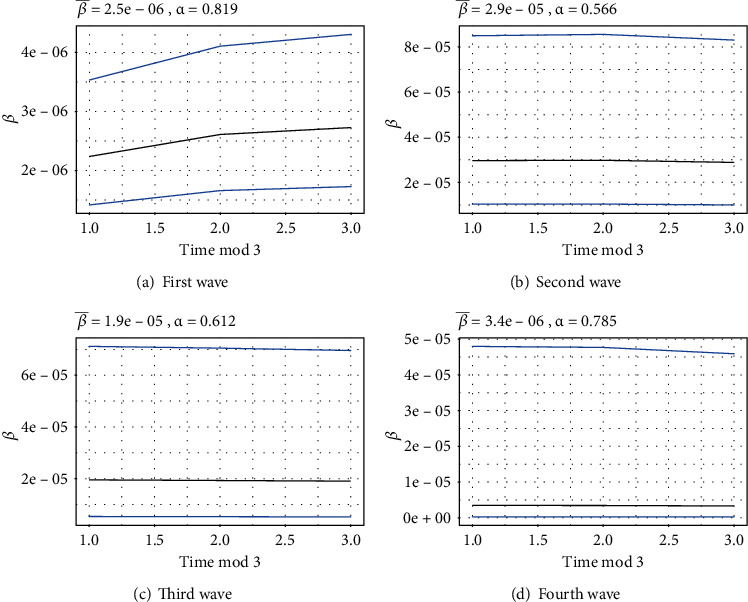
Wave-wise behavior of the contact rate (*β*).

Figure 4 presents the behavior of the contact rate (*β*) along with its respective estimated (by the tSIR model) intervals for four waves of COVID-19, respectively. Furthermore, it represents *α*, which is the disease intensity parameter.

### 3.1. Five-Hundred-Day Analysis

**Figure 5:**
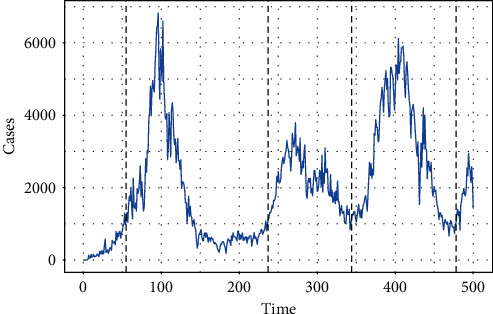
Outbreak plot of the data (500 days).

Figure 5 depicts the daily number of cases for the first five hundred days of COVID-19. One can observe that there are four waves, and it can be observed that the highest number of cases is observed for the first wave. It is worth mentioning that to depict the figure, we set 1000 as the outbreak threshold.

**Figure 6:**
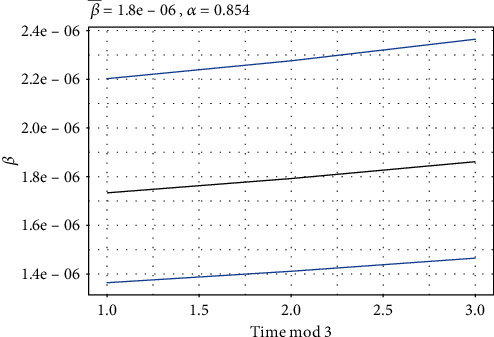
Behavior of the contact rate (*β*) for the first five hundred days of COVID-19.

Figure 6 depicts the evolution of *β* for the first 500 days of COVID-19 data in Pakistan, as well as its estimated intervals. It also reflects the severity of the disease (*α*). The contact rate appears to be growing with time, which may lead to an increase in the daily number of cases.

**Figure 7:**
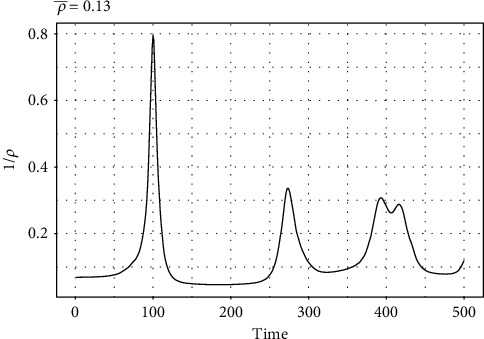
Behavior of the reporting parameter (*ρ*).

The behavior of the estimated reporting rate (*ρ*) using the first 500 days of COVID-19 data for Pakistan is shown in Figure 7. One can observe that in the case of the first wave, there are a less number of cases which are actually positive and not reported as compared to all other waves of COVID-19.

**Figure 8:**
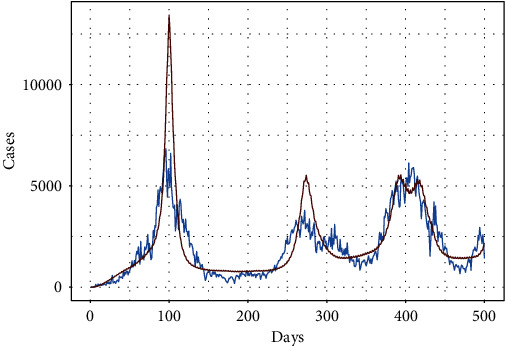
Graph of the fitted tSIR model to the data (500 days).

Figure 8 shows the fitted tSIR model to the data (500 days). The blue curve represents the observed number of daily instances, whereas the gray curve represents the estimated curve using the tSIR model. Furthermore, the predicted number of cases in the middle of the first wave of COVID-19 is extremely high when compared to the observed cases. Similarly, the second-wave model gives an indication of more instances than the observed cases. However, the predicted values after 350 days seem somewhat better than the preceding waves.

Table 2 contains the summary of the mean absolute percentage error (MAPE(*t*)), mean absolute error (MAE(*t*)), and $\bar{\rho }$ for training data ($\bar{\rho }\left(t\right)$) by the tSIR using different distributions for the first 500 days of training data. The MAPE(*f*) and MAE(*f*) for the 10-day projected data are also shown in Table 2. As it can be seen that for the Gaussian distribution with the link “log” in both the training and forecasted (tested) data, the tSIR model performs better. Similarly, Tables 3–6 exhibit the MAPE, MAE, and $\bar{\rho }$ for four waves, respectively. Finally, we conclude that the Gaussian distribution with the link “identity” is appropriate.

Table 1 lists the estimated parameters for 500 days for four waves by the tSIR model.

## 4. Analysis Using the SIR Model

**Figure 9:**
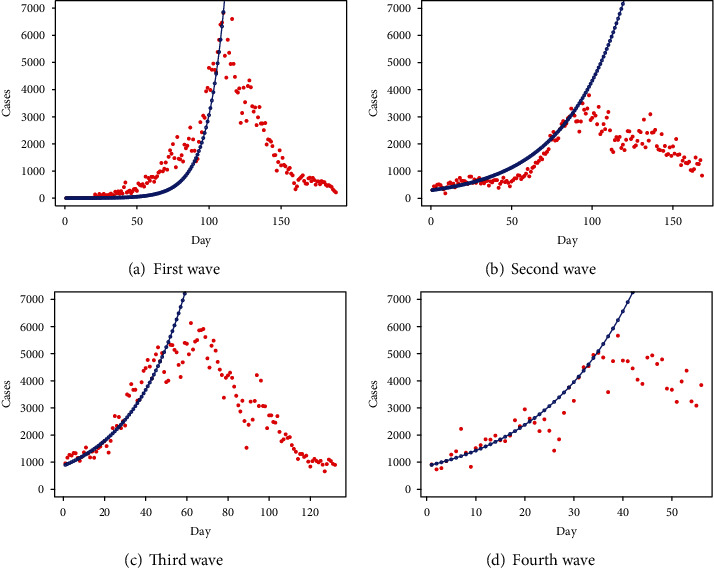
Wave-wise fitted SIR model with the 14-day infectious period.

**Figure 10:**
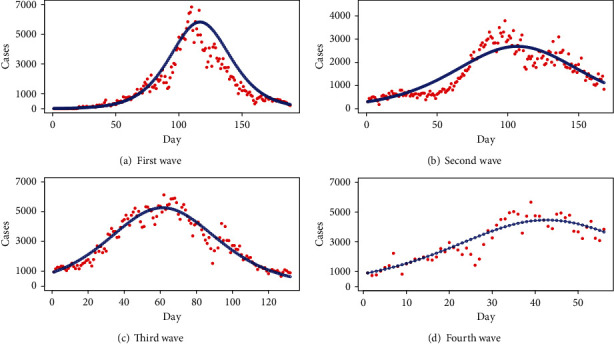
Wave-wise fitted SIR model.

**Figure 11:**
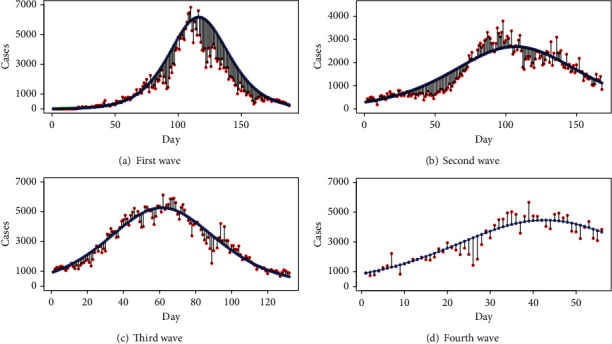
Wave-wise fitted SIR model with errors.

Figure 9 presents the four-wave analysis with the SIR model using the 14-day infectious period and considering the total population of Pakistan as the susceptible population. Contrary to the tSIR model, each wave has a distinct number of starting infected people with rapid exponential growth, indicating that COVID-19 is an epidemic in Pakistan and will infect a large population. This also indicates that the traditional SIR model should not be used for the data.  Figure 10 presents the fitted SIR model for four waves with decreased susceptible population violating the assumption of an infectious period for 14 days, as stated by WHO. The fitted curves are produced using the optimal values of parameters *β* and *γ*, which result in the smallest error sum of squares (ESS). If we assume a 14-day infectious period, the daily number of cases increases dramatically and does not decrease as shown in Figure 9. Furthermore, the contact rate is too low in both situations (with optimal settings and 14-day infectious duration). Thus, we may infer that though disease exists in Pakistan, it is not a pandemic like it is in other nations.  Figure 11 shows the fitted SIR model to wave-wise data with errors.

**Figure 12:**
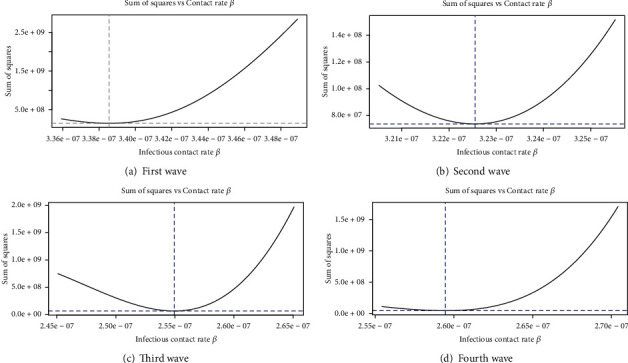
Optimum values of the contact rate (*β*) for four waves.

**Figure 13:**
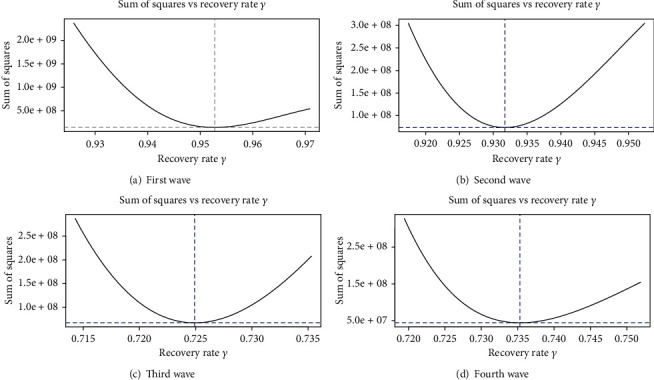
Optimum values of the recovery rate (*γ*) for four waves.

Figure 12 depicts the wave-wise optimum values of the contact rate (*β*) with the minimum residual sum of squares (RSS). It can be seen that the first wave has a higher rate of infectious pathogen interaction than the others. However, this is due to the fact that the government did not take any strict steps against COVID-19 in the beginning. Due to smart and complete lockdowns, pathogen contact rates are minimal in other wave cases. Similarly, Figure 13 depicts the optimum values of the recovery rate (*γ*) for four waves, and it is noticed that the recovery rate (*γ*) is greater in the first wave and steadily decreases in subsequent waves. This happened because individuals used to adhere to the SOPs and scientists also advised antibodies to combat the virus. It is also noticed from the figures reported in the supplementary text that the intensity of illness rises as the pathogen contact rate increased. Also, the ESS increased due to the rise of *β* and *γ*.

Table 7 lists the parameters estimated using the SIR model, and one can observe that *R*_0_ in all waves of COVID-19 in Pakistan is about 1.05, which is a strong indication that COVID-19 is not endemic in Pakistan.

## 5. Analysis Using the Bayesian Approach to the SIR Model

**Figure 14:**
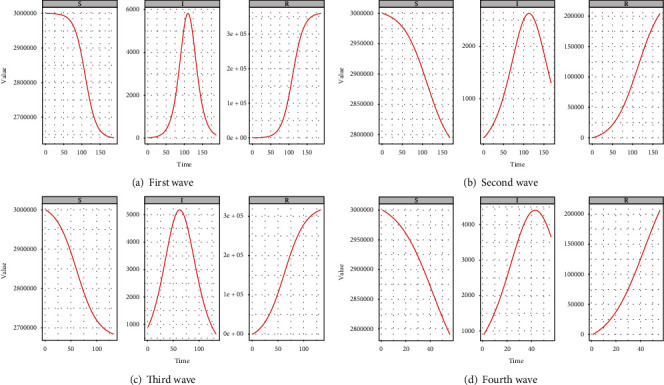
Wave-wise estimated number of susceptible, infected, and recovered people by the pSIR model.

Figure 14(a) depicts the estimated number of susceptible, infected, and recovered people by using the pSIR model for the first-wave data assuming uniform distribution as the prior distribution of*R*_0_ and Poisson distribution for the log-likelihood function. It can be seen that this model behaves very similarly to the traditional SIR model. Here, we used the optimal parameter (estimated from the SIR model) values with the lowest RSS. Furthermore, the overall number of susceptible people is gradually reducing, near 2,600,000 after 188 days. Similarly, the recovery rate is increased with time, approaching 400,000 after 188 days. Furthermore, a large number of everyday instances follow a basic SIR model.  Figure 14(b) shows the estimated number of susceptible, infected, and recovered people for second-wave data using the pSIR model. After 150 days of second-wave data, the overall number of susceptible people is steadily reducing, approaching 2,800,000. Similarly, after 150 days, recoveries exceed 250,000. Furthermore, the 113th day of the second-wave data had the highest number of daily positive cases. Similarly, Figures 14(c) and 14(d) present the estimated behavior of susceptible, infected, and recovered people for the third and fourth waves of COVID-19, respectively.

**Figure 15:**
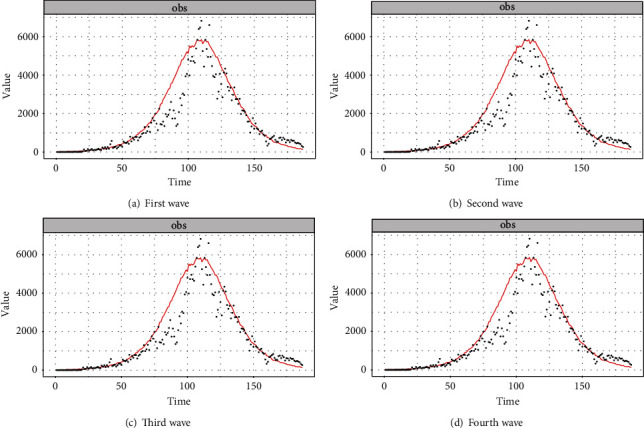
Wave-wise fitted pSIR model to the data.

Figure 15 depicts the wave-wise anticipated number of infected people, and by comparing this figure with Figure 10, one can see that there is no discernible difference between the SIR and pSIR models, except that SIR is a simple mathematical model, whereas the pSIR is a Bayesian SIR model.

Table 8 compares uniform, normal, and Poisson distributions as the prior distribution of*R*_0_ while taking different distributions of log-likelihood on the basis of RSS for each wave of COVID-19 in Pakistan. One can see that in using uniform distribution as the prior distribution of*R*_0_ and Poisson distribution for the log-likelihood, the RSS is the least for all waves as compared to other combinations of distributions.

## 6. Conclusion and Recommendations

In the past, many pandemics have occurred throughout the world, and epidemiological modeling is always an attractive research field in predicting disease dynamics and making optimal decisions. Currently, the deadly COVID-19 pandemic has affected our lives. In this study, we focused on the COVID-19 data from Pakistan and modeled it using epidemiological models. To this end, we used the tSIR model for disease prediction using people with respiratory issues. We observed that the forward simulation of the model for the data was very close to the observed data. It is also discovered that COVID-19-positive cases are underreported using the tSIR model because everyone is frightened of this deadly virus and does not report the COVID-19 test result, and even test reports are biased/forged. As a result, the first estimated number of people who are vulnerable is just 1% of the total population.

Next, we used the two SIR models; one is the traditional model, and the other one is the pSIR model (using uniform distribution as prior distribution of *R*_0_). Taken together, the COVID-19 pandemic characteristics are inconsistent with the SIR modeling paradigm, and the dynamics of this pandemic are influenced by a number of variables. The main reason for this outcome is the lack of reliable data. If we follow the assumptions of the model, the SIR models generate relatively coarse fitted curves, as we saw in the analysis. If we utilize the optimal parameter values, *R*_0_ approaches 1, which means that if we use the fixed infectious period (as determined by WHO), *R*_0_ will be small. This implies that COVID-19 is not a pandemic in Pakistan. One outcome which is often understated is that the simple SIR model, with some minor modifications, does a remarkably good job at predicting the size, extent, and shape of a single wave. That is why the many more sophisticated extensions of SIR all seem to largely agree, which has been a great advantage for the health response teams. By the middle of the first wave, our hospitals were overrun with infections, and thus, without a question mark, the Pakistani government has responded quickly to this deadly virus by implementing clever and full lockdowns, public awareness campaigns, quarantines, and screening centers. To combat future pandemics, we recommend that the government establish additional hospitals at each district level. In addition, there should be a secure and proper data reporting mechanism.

There are several problems in this study that have gone unnoticed. For example, we did not use vaccination data and avoided the cases that were exposed. Only the persons with respiratory issues were utilized as a variable connected to the daily number of cases in this study. Thus, the tSIR model may be studied with “daily deaths” as a primary variable. Furthermore, the extended SIR models may be used to examine wave-by-wave data.

## Figures and Tables

**Table 1:** Estimates of the tSIR model using data (500 days) and wave-wise data.

Parameters	Data	First wave	Second wave	Third wave	Fourth wave
$\bar{\beta }$	1.80*E*-06	2.53*E*-06	2.94*E*-05	1.93*E*-05	3.39*E*-06
$\bar{\rho }$	0.1290	0.1070	0.0980	0.1740	0.1460
$\bar{S}$	2.29*E*+06	2.24*E*+06	2.27*E*+06	2.29*E*+06	2.27*E*+06
*S*_0_	7.79*E*-04	8.61*E*-03	9.97*E*-03	1.00*E*-02	1.00*E*-02
*I*_0_	6.53*E*-08	1.58*E*-07	2.10*E*-05	3.96*E*-05	3.68*E*-05
*α*	0.8050	0.8200	0.5700	0.6100	0.7900

**Table 2:** MAPE and MAE using the first 500 days as training data and 10 days as testing data using different distributions.

Distributions	Link	MAPE(*t*)	MAE(*t*)	$\bar{\rho }\left(t\right)$	MAPE(*f*)	MAE(*f*)
Quasi-Poisson	Log	38.09	619.71	0.13	18.5862	650.3652
Poisson	Log	38.16	621.04	0.13	18.7826	651.3254
Gaussian	Log	36.58	617.39	0.13	17.9658	643.2589
Gaussian	Identity	36.95	620.27	0.13	17.4062	640.2154

**Table 3:** MAPE and MAE of the first wave using different distributions.

Distributions	Link	MAPE	MAE	$\bar{\rho }$
Quasi-Poisson	Log	65.92	300.89	0.11
Poisson	Log	65.31	300.58	0.11
Gaussian	Log	79.39	305.72	0.11
Gaussian	Identity	30.32	288.94	0.11

**Table 4:** MAPE and MAE of the second wave using different distributions.

Distributions	Link	MAPE	MAE	$\bar{\rho }$
Quasi-Poisson	Log	14.73	190.36	0.098
Poisson	Log	14.74	190.26	0.098
Gaussian	Log	14.81	190.67	0.098
Gaussian	Identity	14.25	188.79	0.098

**Table 5:** MAPE and MAE of the third wave using different distributions.

Distributions	Link	MAPE	MAE	$\bar{\rho }$
Quasi-Poisson	Log	13.04	335.08	0.17
Poisson	Log	13.05	335.51	0.17
Gaussian	Log	13.05	335.44	0.17
Gaussian	Identity	12.85	334.22	0.17

**Table 6:** MAPE and MAE of the fourth wave using different distributions.

Distributions	Link	MAPE	MAE	$\bar{\rho }$
Quasi-Poisson	Log	33.13	730.06	0.15
Poisson	Log	33.02	727.96	0.15
Gaussian	Log	33.43	733.96	0.15
Gaussian	Identity	33.01	704.81	0.15

**Table 7:** Wave-wise estimated parameters of the SIR model.

Parameters	First wave	Second wave	Third wave	Fourth wave
*β*	3.448483*E*-7	2.65091*E*-7	2.8442*E*-7	4.26428*E*-7
*γ*	0.9709	0.7637	0.8079	1.2175
RSS	116,470,946	27,059,876	29,087,890	19,050,557
*R*_0_	1.0656	1.0413	1.0561	1.0507

**Table 8:** Comparison of different prior and likelihood distributions of *R*_0_.

Prior distribution	Log-likelihood distribution	Waves	*R*_0_	*P*_inf_	RSS
Uniform	Poisson	1	1.0656	0.9709	**116,725,361**
		2	1.0413	0.7637	**29,235,687**
		3	1.0561	0.8080	**30,256,324**
		4	1.0507	1.2175	**21,548,964**
Normal	Normal	1	1.0656	0.9709	119,623,567
		2	1.0413	0.7637	30,887,555
		3	1.0561	0.8080	31,998,541
		4	1.0507	1.2175	21,999,219
Poisson	Poisson	1	1.0656	0.9709	121,938,652
		2	1.0413	0.7637	31,658,742
		3	1.0561	0.8080	32,526,487
		4	1.0507	1.2175	23,145,985

## Data Availability

The dataset used in this article is available at https://ncoc.gov.pk/.

## Supplementary Materials

Supplementary Materials The supplementary text contains the additional tables and figures.
