# Supplementary material for: COVID-19 Pandemic Data Modeling in Pakistan Using Time-Series SIR
Source: Comput Math Methods Med. 2022 Jun 28;2022:6001876. doi: 10.1155/2022/6001876 (PMC9256309; doi:10.1155/2022/6001876)
Supplement: Supplementary Materials — The supplementary text contains the additional tables and figures. [file 6001876.f1.pdf]

SUPPLEMENTARY TEXT: COVID-19 PANDEMIC DATA MODELING IN PAKISTAN USING TIME SERIES SIR

Muhammad Taimoor[1], Sajid Ali[*,2], Ismail Shah[3], and Fred Roland Muwanika[*,4]

[1,2,3] *Department of Statistics, Quaid-i-Azam University, Islamabad, 45320, Pakistan*

[4] *School of Statistics, Makerere University, P. O. Box 7062, Kampala, Uganda*

[1] *taimoorm1995@gmail.com*, [*,2] *sajidali.qau@hotmail.com*, [3] *ishah@qau.edu.pk*,

[*,4] *frmuwanika@yahoo.co.uk*

**Corresponding Authors*

Abstract. Pakistan is currently facing fourth wave of the deadly corona virus, which was first reported in Wuhan, China in December 2019. This work utilizes the epidemiological models to analyse Pakistan's COVID-19 data. The basic susceptible, infected, and recovered (SIR) model is studied assuming Bayesian and time series SIR (tSIR) approaches. Many studies have been conducted from different perspectives, but to the best of our knowledge no study is available using the SIR models for Pakistan. The corona virus incubation period has been set to 14 days across the globe, however, this study noticed that the assumption of 14 days is not suitable for Pakistan data. Furthermore, on the basis of R_0, we infer that COVID-19 is not a pandemic in Pakistan, as it was in other nations, such as the United States, India, Brazil, Italy, among others. We attribute this to the best strategy adopted by the Government of Pakistan to minimize the burden of COVID-19 cases in Pakistani hospitals. It is also noticed that the posterior based SIR (pSIR) model with uniform prior for R_0 and Poisson distribution (of log-likelihood) provides better results as compared to other distributions. From time series SIR (tSIR), we observed that the value of reporting rate (ρ) is less than 1 which means that cases are under-reported.

Keywords: COVID-19, Time Series SIR, Susceptible, Compartment Modeling, Bayesian SIR

1. Exploratory data analysis

This section presents the exploratory data analysis of COVID-19 data taken from `https://covid.gov.pk/stats/pakistan`. In particular, we focus on three main variables (daily new cases, daily new deaths, and daily recoveries). Currently, Pakistan is facing the fourth wave of the COVID-19, thus we present the wave-wise analyses too.

Tables S.1-S.5 present the wave-wise descriptive summaries. In particular, the mean number of daily cases, deaths, and recoveries are 2033.91, 45.29 and 1806.83, following by 67.58, 7.96 and 83.79 standard errors, respectively. Furthermore, the maximum number of cases, 6825, occurred in the first wave of the pandemic. Similarly, the maximum deaths are 201, which were observed in the third wave. Total 1086108, 24187 and 964849 number of cases, deaths, and recoveries were reported till August 12, 2021. From Table S.2, one can see that the mean number of daily cases is 1573.75 with 122.44 standard error. The first outbreak period was from February 26, 2020 to August 31, 2020 with 6825, 153 and 16813, as the maximum number of daily cases, deaths and recoveries, respectively. There were total 295866 positive cases, 6294 deaths, and 280682 recoveries reported in the first outbreak of the COVID-19.

One can see from the descriptive analysis of the second-wave of COVID-19 reported in Table S.3, the spell of the deadly virus was from September 1, 2020 to February 16, 2021, the maximum number of deaths (in one day) in the second-wave was 111, followed by 3795 and 13932 maximum daily cases and recoveries, respectively. Similarly, the third wave started on February 17, 2021 and ended on June, 23, 2021 is given in Table S.4. The fourth-wave of the COVID-19 started from June 24, 2021 to date and the summary is given in Table S.5.

2. Graphical analysis

This section comprises the graphical presentation of the data under study. More specifically, the graphical presentation of the daily confirmed, daily deaths, and daily recoveries of COVID-19 patients in Pakistan is given S.1. Boxplots depicted in Figure S.1 and Table S.6

Table S.1. Descriptive analysis of the data

**Descriptive statistics**	**Daily cases**	**Daily deaths**	**Daily recoveries**
Mean	2033.9101	45.2940	1806.8333
Standard Error	67.8534	7.9624	83.7901
Median	1640.5	39	1298.5
Mode	1	0	0
Standard Deviation	1567.9870	183.9991	1936.2590
Sample Variance	2458583.3350	33855.7051	3749099.0210
Kurtosis	-0.1985	244.0397	14.2658
Skewness	0.8115	-0.5172	2.8901
Range	6824	5877	16813
Minimum	1	0	0
Maximum	6825	201	16813
Sum	1086108	24187	964849
Count	500	500	500
Confidence Level(95.0%)	133.2929	15.6415	164.5993

Table S.2. Descriptive analysis of **First-wave** data

**Descriptive statistics**	**Daily cases**	**Daily deaths**	**Daily recoveries**
Mean	1573.7553	33.4787	1492.9893
Standard Error	122.4434	2.5882	177.1822
Median	841.5	20	617
Mode	1	0	0
Standard Deviation	1678.8606	35.4884	2429.4012
Sample Variance	2818572.9450	1259.4277	5901990.5770
Kurtosis	0.7428	0.8089	14.4148
Skewness	1.2678	1.2224	3.3400
Range	6824	153	16813
Minimum	1	0	0
Maximum	6825	153	16813
Sum	295866	6294	280682
Count	188	188	188
Confidence Level(95.0%)	241.5480	5.1059	349.5330

of five number summary of the three categories of data show that there are a small number of extreme values in confirmed cases and a large number of extreme values in the recoveries (including some outliers as well). Also, the daily confirmed cases are slightly more dispersed as compared to the daily recoveries, whereas the daily deaths data is void of such attributes. Figure S.2 depicts the histograms of the three categories shows that all waves have positively

Table S.3. Descriptive analysis of **Second-wave** data

Discriptive statistics	Daily cases	Daily deaths	Daily recoveries
Mean	1595.3392	36.0119	1460.2083
Standard Error	71.2172	2.4973	116.9420
Median	1578.5	33.5	1206
Mode	736	6	489
Standard Deviation	923.0808	32.3687	1515.7422
Sample Variance	852078.2614	1047.7363	2297474.6690
Kurtosis	-1.1389	23.2914	28.7564
Skewness	0.3162	3.2670	4.2691
Range	3614	294	13814
Minimum	181	1	118
Maximum	3795	111	13932
Sum	268017	6050	245315
Count	168	168	168
Confidence Level(95.0%)	140.6021	4.9303	230.8753

Table S.4. Descriptive analysis of **Third-wave** data

Descriptive statistics	Daily cases	Daily deaths	Daily recoveries
Mean	3039.1496	76.3858	2900.2755
Standard Error	139.0332	3.3192	129.9670
Median	2869	71	2631
Mode	1050	118	4123
Standard Deviation	1566.8248	37.4057	1464.6540
Sample Variance	2454940.1920	1399.1912	2145211.6323
Kurtosis	-1.3160	-0.0541	-0.7861
Skewness	0.2165	0.7074	0.4725
Range	5464	185	6097
Minimum	663	16	752
Maximum	6127	201	6849
Sum	385972	9701	368335
Count	127	127	127
Confidence Level(95.0%)	275.1425	6.5686	257.2009

skewed distributions meaning that a large number of values are concentrated on the left side of the distribution.

3. tSIR Model Analysis

Figure S.3 shows the behavior of the daily reported COVID-19 cases in Pakistan. In particular, Figures S.3a, S.3b, S.3c and S.3d are depicted for four waves to assess the behavior

Table S.5. Descriptive analysis of **Fourth-wave** data

Descriptive statistics	Daily cases	Daily deaths	Daily recoveries
Mean	2671.6274	42.2156	1382.6862
Standard Error	208.2001	3.0735	126.6488
Median	2228	37	1084
Mode	1980	40	1110
Standard Deviation	1486.8465	21.9493	904.4533
Sample Variance	2210712.7983	481.7725	818035.8996
Kurtosis	-1.2358	0.4044	8.0737
Skewness	0.4289	1.0495	2.7373
Range	4926	91	4375
Minimum	735	11	650
Maximum	5661	102	5025
Sum	136253	2153	70517
Count	51	51	51
Confidence Level(95.0%)	418.1824	6.1733	254.3816

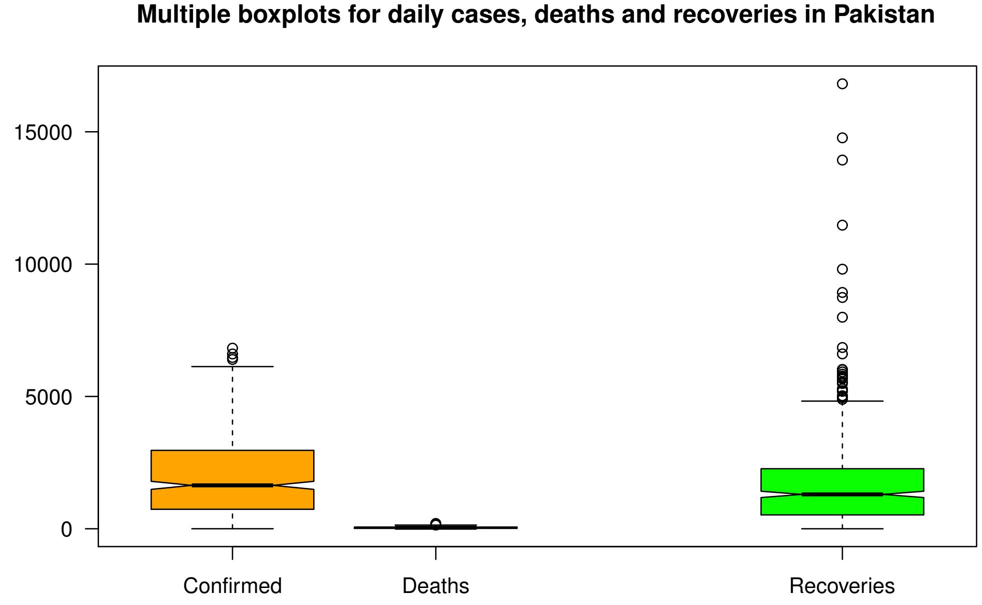

Figure S.1. Boxplot of daily confirmed, deaths, and recoveries in Pakistan

of the daily number of reported cases of COVID-19 in Pakistan. Figure S.3a depicts that there are low reported cases at the start of the first wave, which increased exponentially while after some days they exponentially decay. Similarly, Figures S.3b, S.3c present the number of daily reported cases for second and third wave, respectively. One can observe

TABLE S.6. Five number summary of variables under study.

Fivenum	Cases	Deaths	Recoveries
Min	1	0	0
Q1	736	15	524
Q2	1640.5	39	1298.5
Q3	2963	65	2268
Max	6825	201	16813

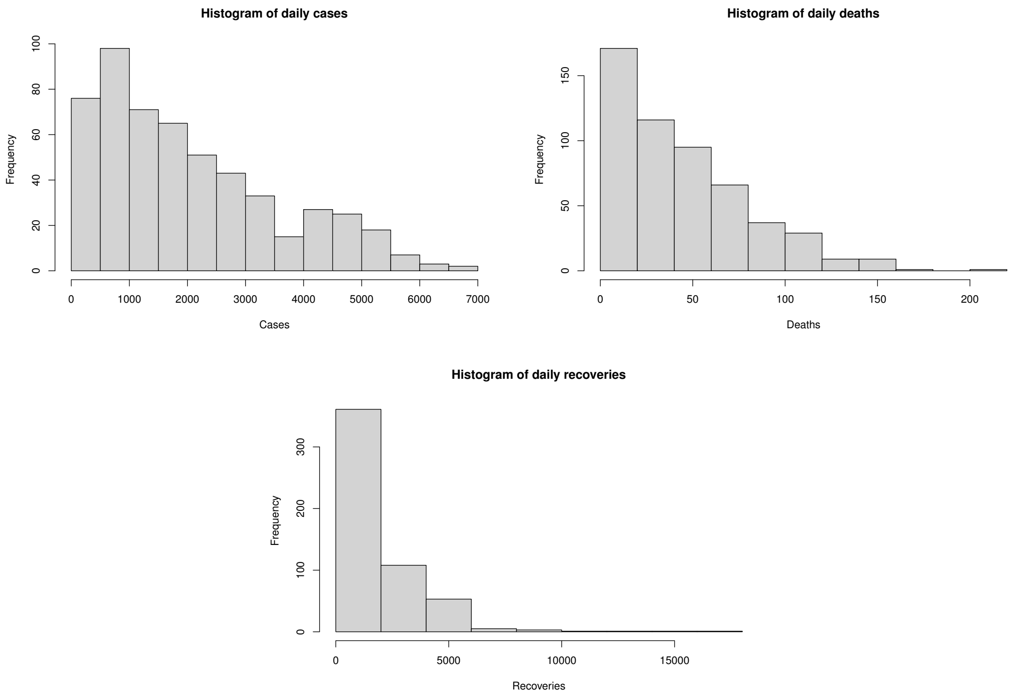

FIGURE S.2. Histograms of the study variables

that the rate of decay for the cases after some days is low as compared to the first wave. Furthermore, Figure S.3d presents the behavior of daily reported cases during the fourth wave of COVID-19, where one can observe that, on average, the cases are increasing with the passage of time and have a monotonic relationship with the time.

Figures S.4a, S.4b, S.4c and S.4d show the actual inverse of daily reported cases (blue curve) and the behavior of cases by the tSIR model (gray curve).

Figures S.5a, S.5b, S.5c and S.5d present the GLM model fitted across four different waves of COVID-19. In each graph, there are three different curves; the red curve presents the

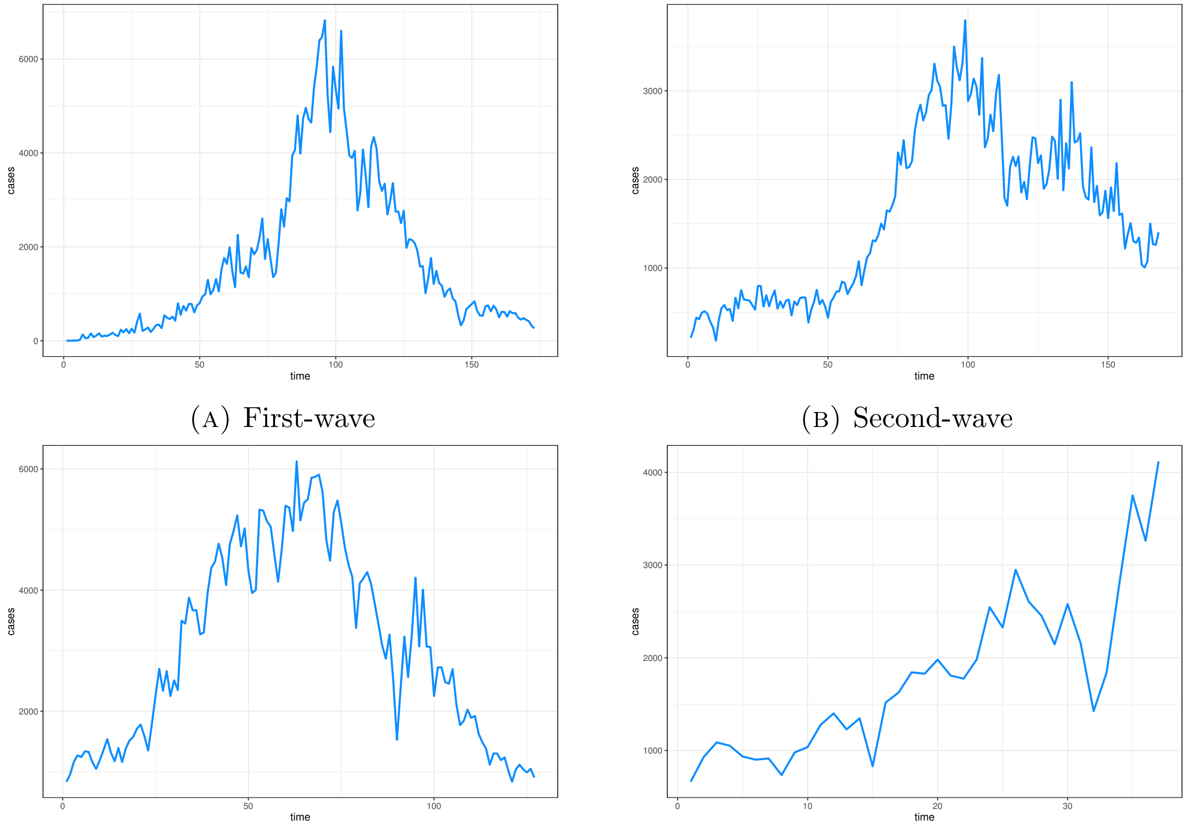

(A) First-wave (B) Second-wave

(C) Third-wave (D) Fourth-wave

FIGURE S.3. Wave-wise Plots of Daily Reported Cases of COVID-19 in Pakistan

daily cumulative number of reported cases, the green curve shows the cumulative people having the respiratory problems while the blue curve shows the GLM fit.

The residuals estimated by tSIR model is shown in Figure S.6 for four waves of COVID-19. One can observe that the error terms are non-random in all waves.

Figures S.7 depicts the behavior of autocorrelation (ACF) for four waves of COVID-19 of Pakistan. One can observe that residuals are not white noise in all the waves and thus model can be further improved if additional covariates are available.

The fitted GLM to the cumulative positive cases and cumulative number of persons with respiratory diseases over the first 500 days of COVID-19 data is shown in Figure S.8. In particular, the red curve is lower than the other two curves, indicating that the daily number of instances are lower than the number of persons with respiratory issues. Then, one may

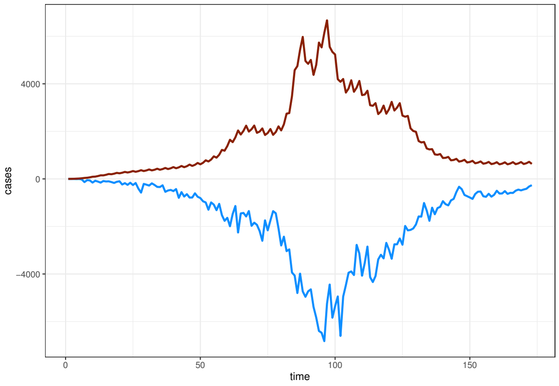

(A) First-wave

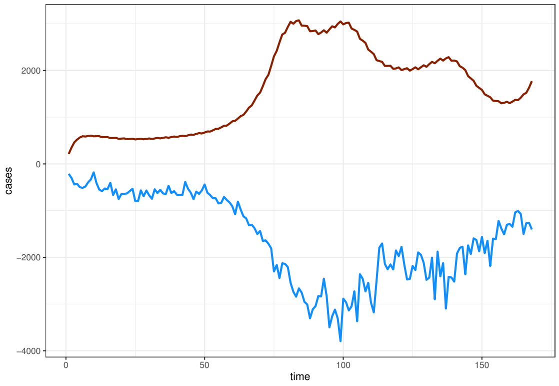

(B) Second-wave

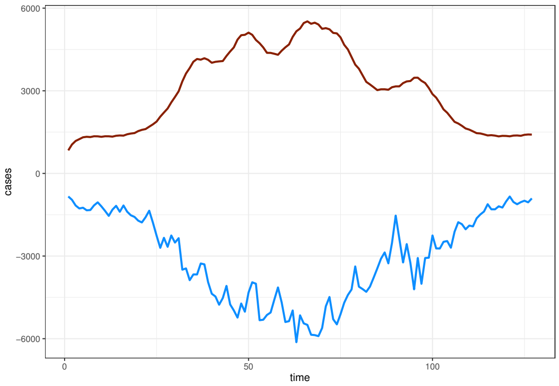

(C) Third-wave

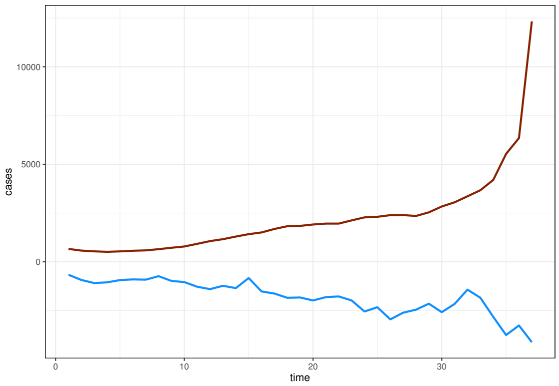

(D) Fourth-wave

FIGURE S.4. Wave-wise Inversely Fitted tSIR Model

argue that the daily number of COVID-19 cases are under reported, because the symptoms of COVID-19 are nearly identical to those of respiratory problems. Figure S.9 presents the effect of the initial number of susceptible with respect to log-likelihood function, where $\bar{S}$ on the x-axis and log-likelihood on the y-axis. One can see that by increasing the mean number of susceptible, the log-likelihood also increased. This means that the estimated mean number of the susceptible is appropriate for the initial values of susceptible, where the log-likelihood is the minimum. This initial number of susceptible is almost 1% of the total population. Figure S.10 presents the inversely fitted model to the data for a better view. Figure S.11 depicts the residuals plot of the data (500 days), which are estimated by the tSIR model. The variance is not uniform throughout the waves, despite the fact that the mean of the error is zero. As a result, the errors are not considered white noise.

Figure S.12 plots the ACF of the errors. One can see that the ACF decays slowly with a predictable pattern. Furthermore, the ACF surpassed the thresholds on virtually all of the lags, indicating that the errors are not white noise. We also used the Box-Ljung test to see whether the errors are white noise or not and the result are numerically presented in Table S.7. Because the p-value is too low, we infer that the errors are not white noise. Table S.7 lists the results of the Box-Ljung test for the errors produced by the tSIR model for data and all waves individually.

4. SIR Model Analysis

Figure S.13 presents the virus intensity for four waves and it can be noticed from sub-figure S.13a that the intensity of illness can rise as the pathogen contact rate increased. Similarly, the patterns of β and γ in the other three waves show a rise or decrease in the

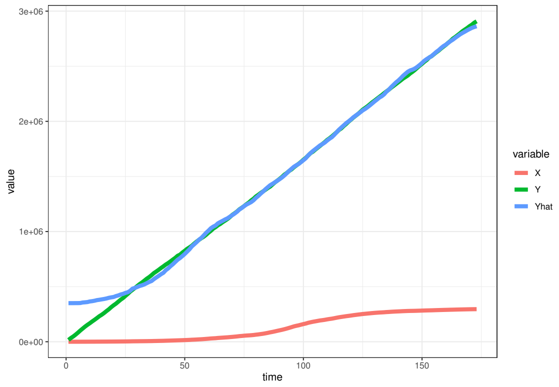

(A) First-wave

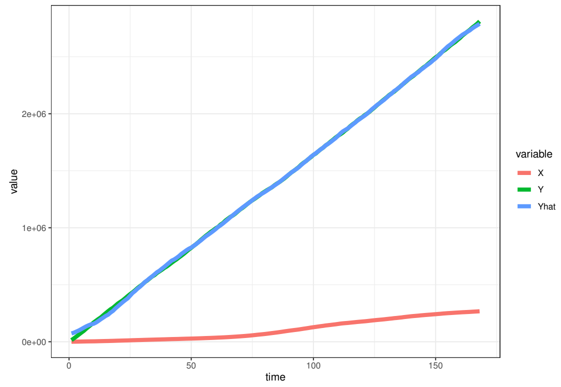

(B) Second-wave

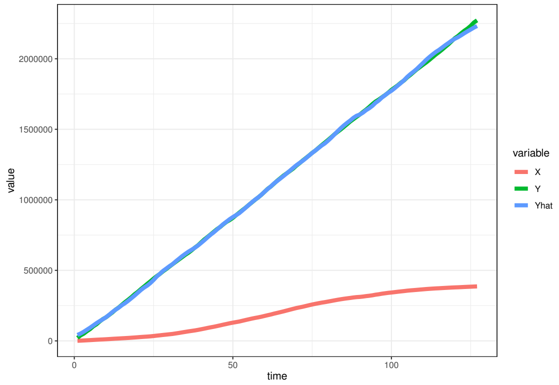

(C) Third-wave

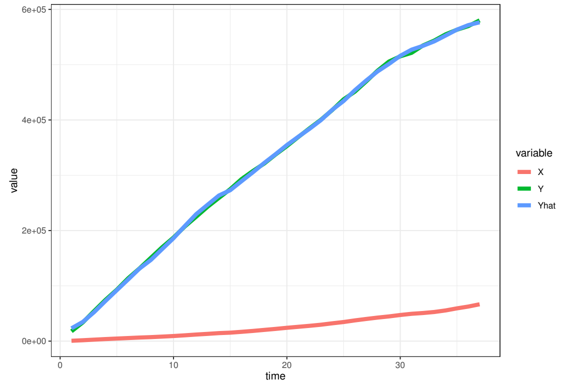

(D) Fourth-wave

Figure S.5. Wave-wise Fitted Regression Model

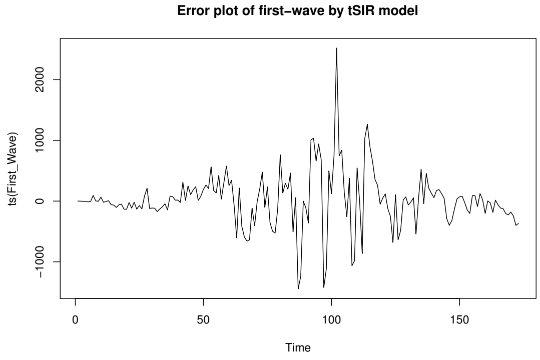

(A) First-wave

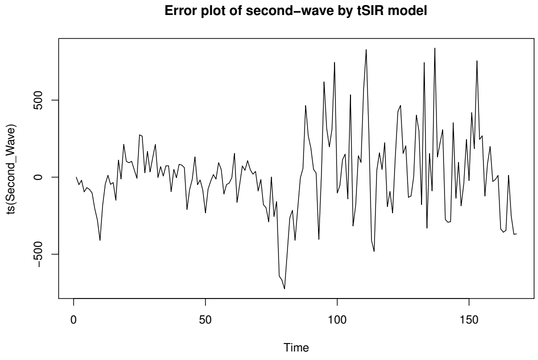

(B) Second-wave

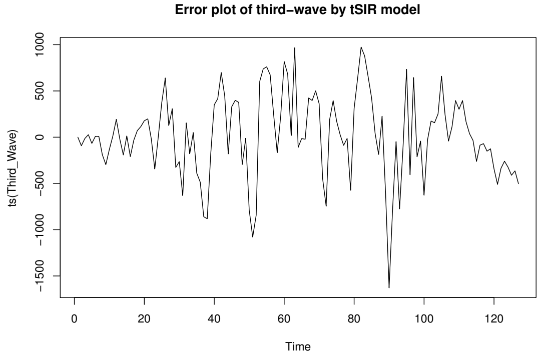

(C) Third-wave

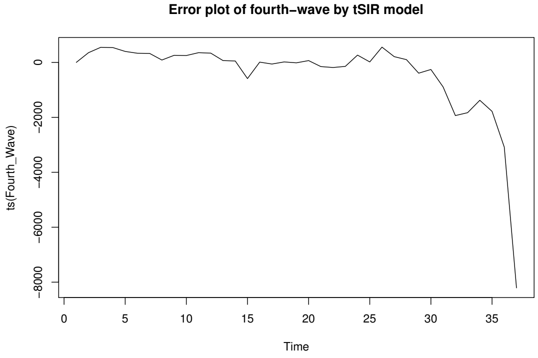

(D) Fourth-wave

FIGURE S.6. Wave-wise Time-series Error Plots Estimated by tSIR Model

TABLE S.7. Box-Ljung test output of data and waves

Data	X-square	df	p-value
**Data (500 days)**	783	2	2.20E-16
**First-wave**	34.997	2	2.52E-08
**Second-wave**	40.551	2	1.57E-09
**Third-wave**	40.656	2	1.49E-09
**Fourth-wave**	12.252	2	2.19E-03

disease intensity. Figure S.14 exhibits the wave-wise behavior of error sum of squares (ESS) against different values of β and γ. In all waves, the ESS increased due to a arise in β and γ.

Figure S.15 depicts the number of susceptible, infected, and recovered by using 14 days as the infectious period. It can be seen that the blue curve represents vulnerable individuals, whereas the red and green curves represent infected and recovered individuals. With a fixed infection time (14 days), we used the optimal value of parameter β. Furthermore, the model returns an almost zero number of infected from the commencement of the disease until the

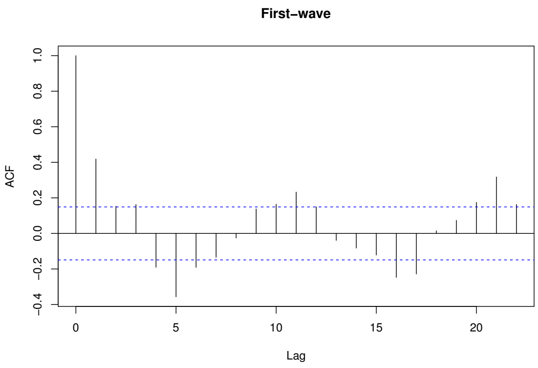

(A) First-wave

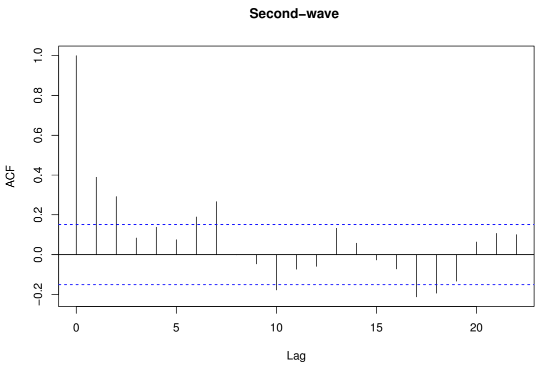

(B) Second-wave

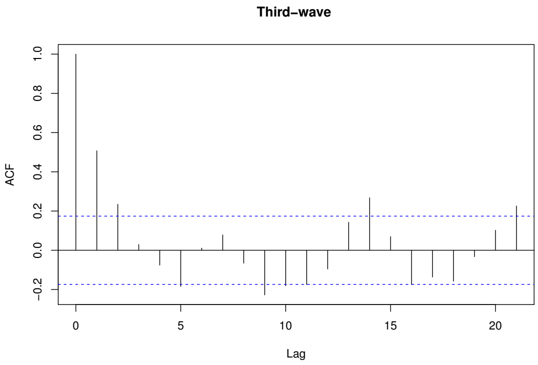

(C) Third-wave

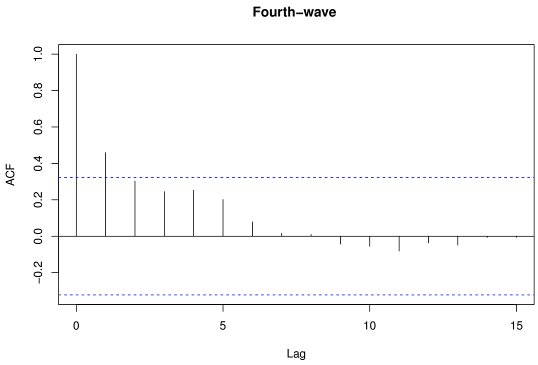

(D) Fourth-wave

Figure S.7. Wave-wise ACF

first 200 days. The greatest number of positive cases (192708) occurred on the 381-day of COVID-19, as shown in Figure S.16, which shows the estimated number of infected persons using the SIR model. On the other hand, the number of confirmed cases each day did not surpass 7000. As a result we infer that the SIR model is not appropriate for this type of disease (which follows a certain pattern), as seen in Figure S.17. The SIR model can be used to predict individual waves, but not for Pakistan because COVID-19 is not a pandemic here. If someone applied the SIR model for individual waves, he would be violating the assumption of an infectious period that is nearly constant across the world.

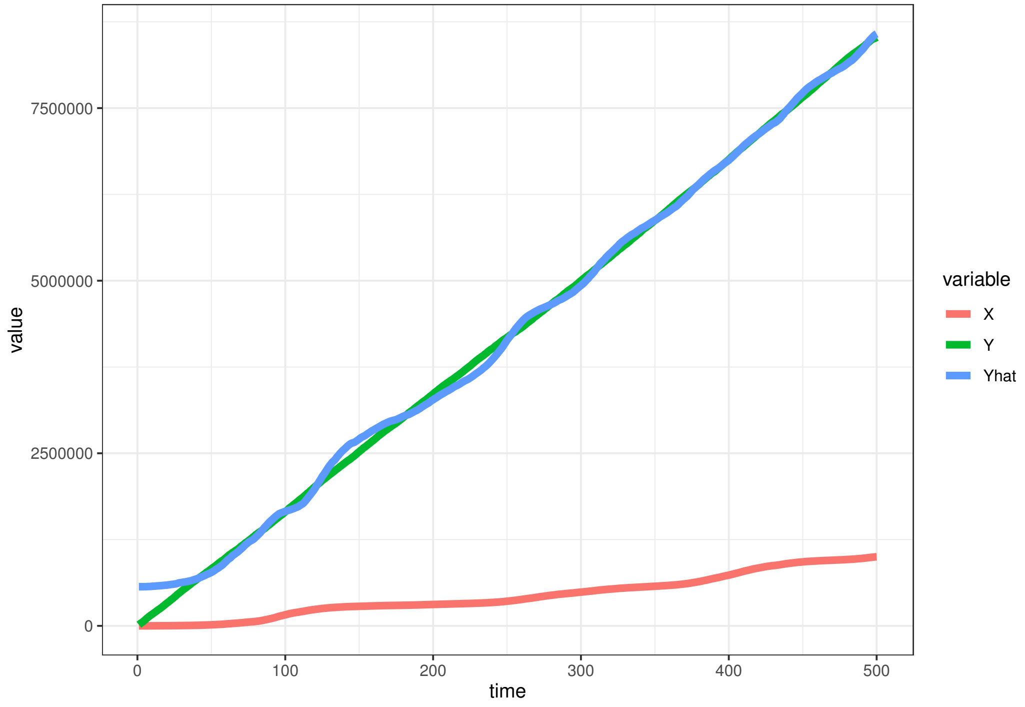

FIGURE S.8. GLM fitted to daily number of cumulative cases and cumulative number of people having respiratory problems

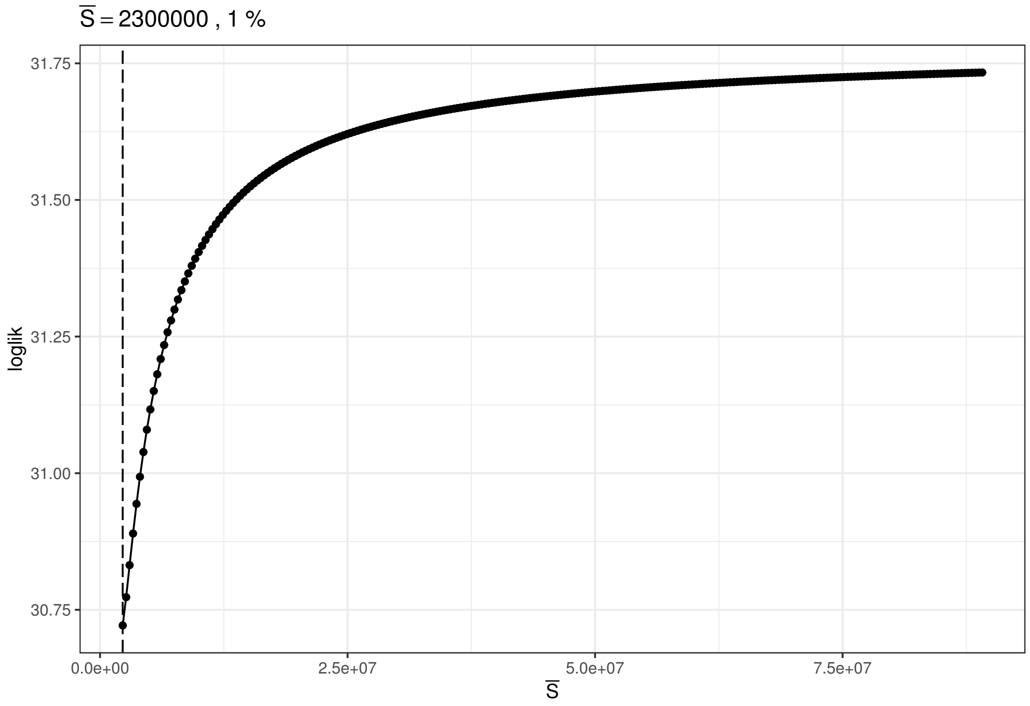

FIGURE S.9. Behavior of mean number of susceptible ($\bar{S}$) estimated by tSIR model

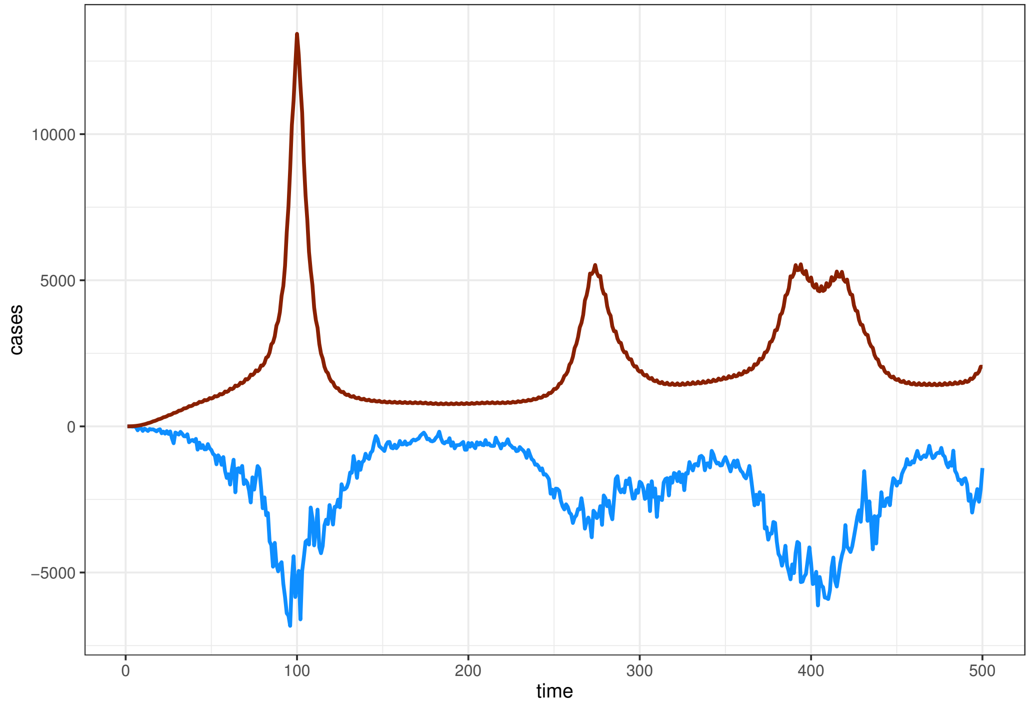

FIGURE S.10. Graph of inversely fitted tSIR model to the data (500 days).

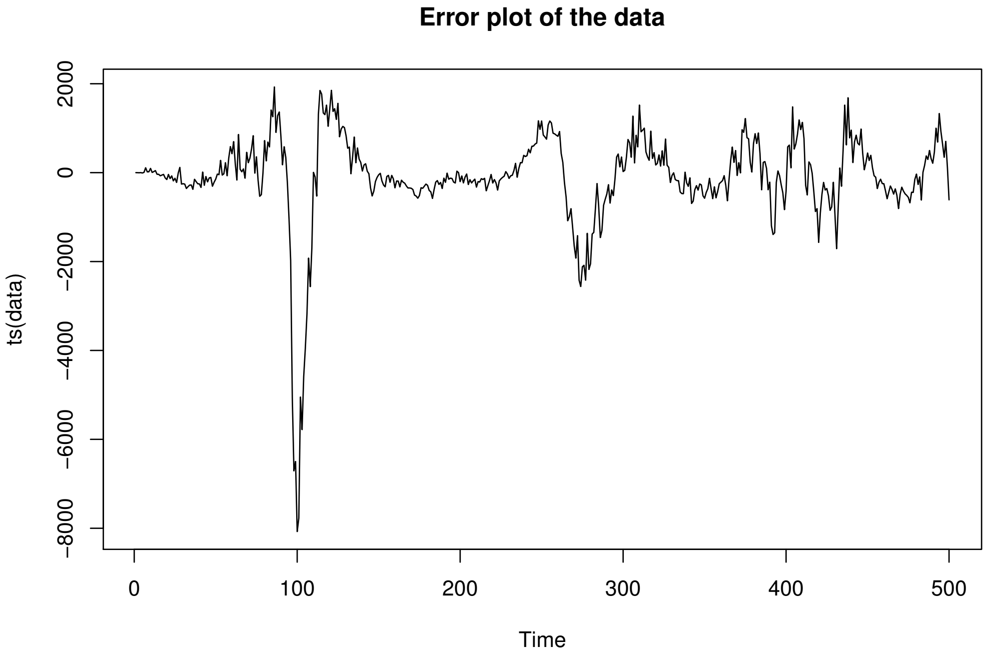

FIGURE S.11. Time series plot of errors estimated by tSIR model

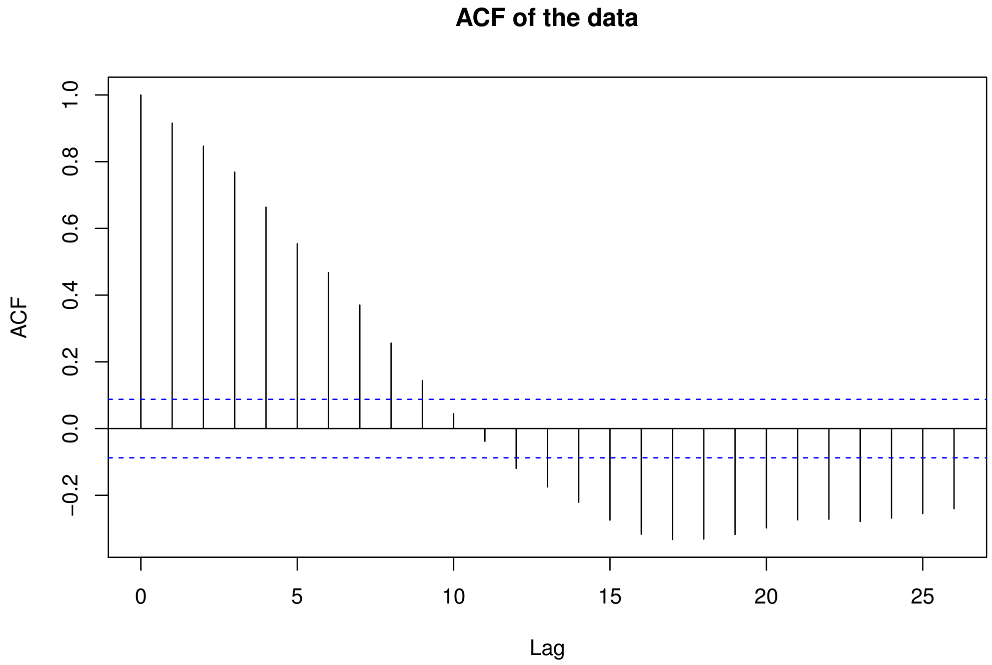

FIGURE S.12. ACF plot of the errors estimated by tSIR model

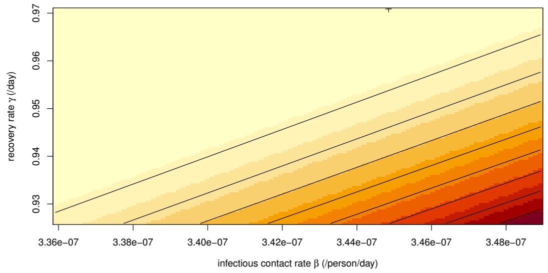

(A) First-wave

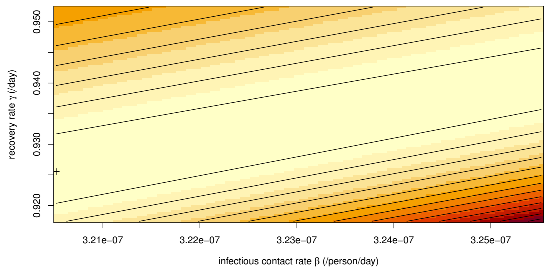

(B) Second-wave

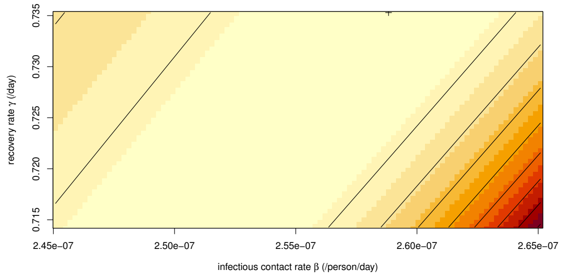

(C) Third-wave

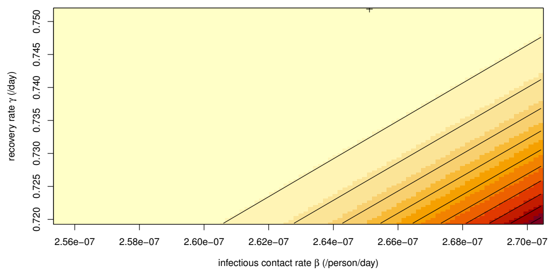

(D) Fourth-wave

FIGURE S.13. Incidence intensity plot of four waves using β and γ

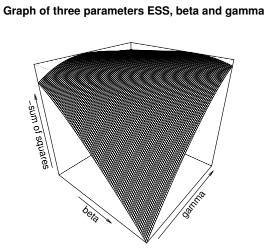

(A) First-wave

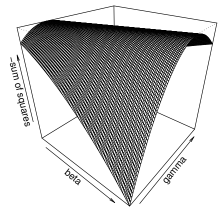

(B) Second-wave

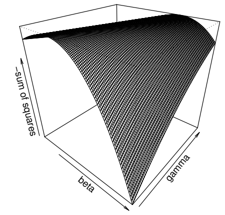

(C) Third-wave

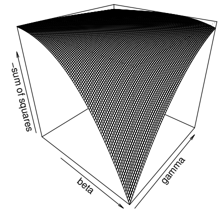

(D) Fourth-wave

FIGURE S.14. Behavior of sum of square against β and γ

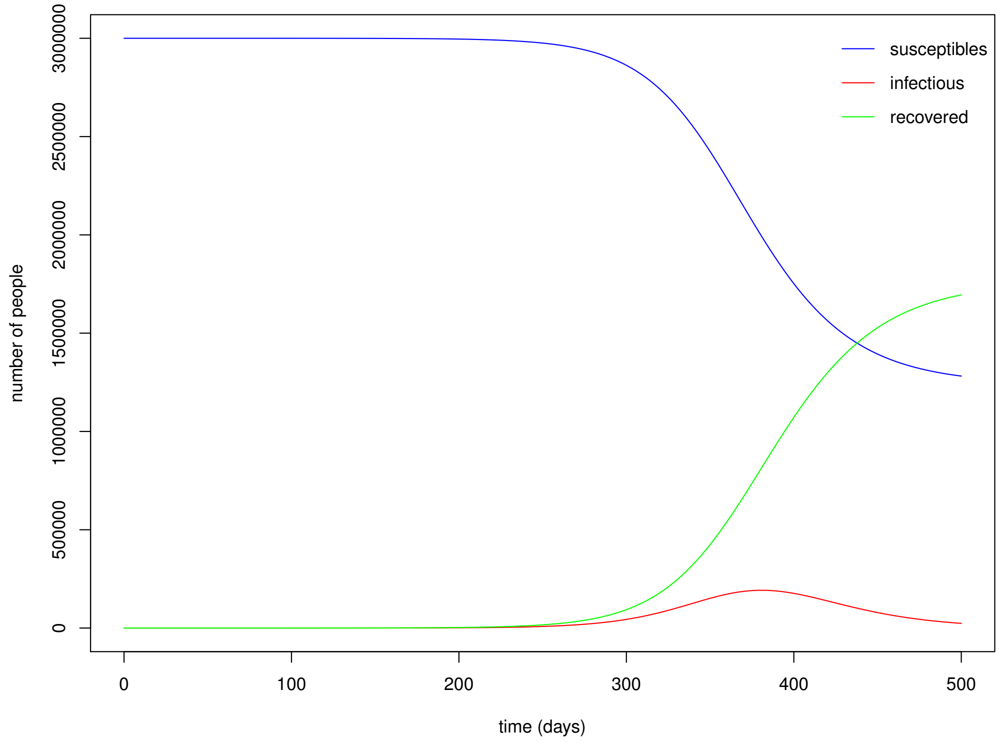

Figure S.15. Number of susceptible, infected, and recovered people using SIR model with 14 days as the infectious period

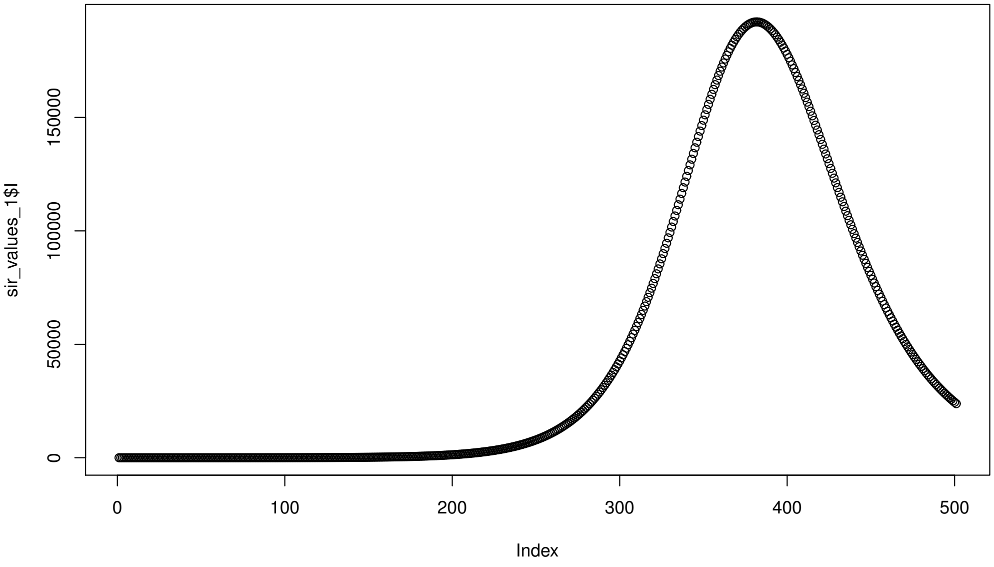

FIGURE S.16. Infected number of people by the SIR model using 14 days as infectious period

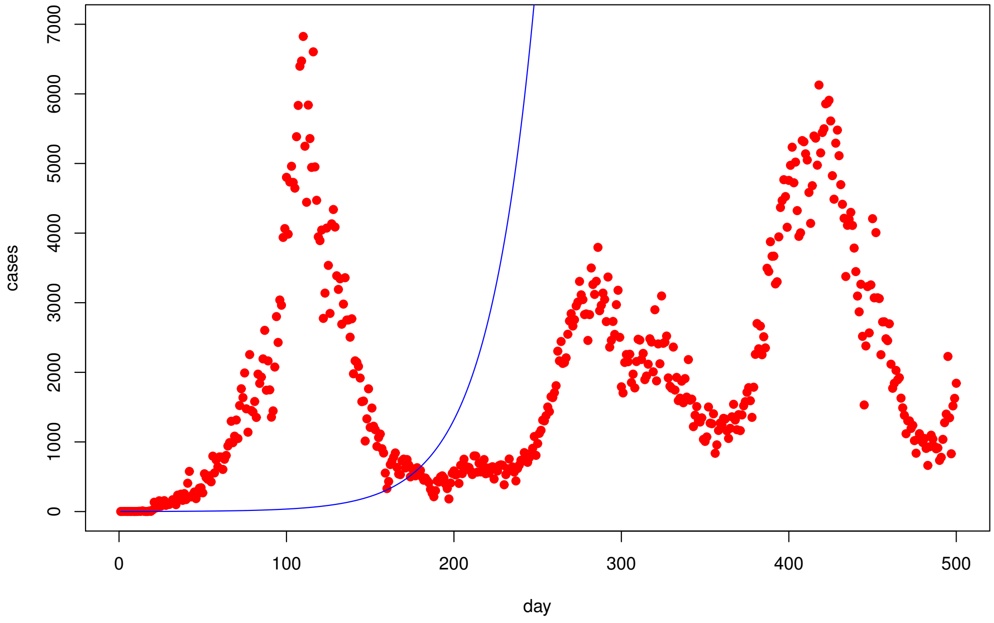

FIGURE S.17. Fitted SIR model to the data (500 days only)
